# Data on low-molecular weight proteins of *Escherichia coli* treated by essential oils components, tetracycline, chlorine and peroxide by MALDI-TOF MS

**DOI:** 10.1016/j.dib.2018.10.050

**Published:** 2018-10-23

**Authors:** Matěj Božik, Pavel Cejnar, Petr Maršík, Pavel Klouček

**Affiliations:** a Czech University of Life Sciences, Faculty of Agrobiology, Food and Natural Resources, Department of Food Science, Kamycká 129, 16500 Prague, Czech Republic; b University of Chemistry and Technology, Department of Computing and Control Engineering, Technická 5, 166 28 Prague 6, Czech Republic

## Abstract

In this dataset we provide MALDI-TOF MS spectra of Escherichia coli. The data presented in this article are related to the article entitled “Stress response of *Escherichia coli* to essential oil components – insights on low-molecular-weight proteins from MALDI-TOF” (Božik et al., 2018) [1]. Essential oils and their components are known for their antibacterial effect. Many studies evaluated the effect of essential oil components (EOCs) on the cell wall, bacterial membranes, and energetic metabolism. But data about low molecular weight proteins (<20 kDa) are limited. Provided data are focused on bacterial response to EOCs; tetracycline, peroxide and chlorine was used as control as common antibiotic and disinfectant agents used against bacteria. These data describe the effect of tested substances to bacterial protein synthesis.

**Specifications table**

**Table:** 

Subject area	*Biology*
More specific subject area	*Microbiology and proteomics*
Type of data	*Table, figure, MS spectra in text files*
How data was acquired	*MALDI-TOF Autoflex Speed (Bruker,DE), flexControl 3.4 (Build 135)*
Data format	*Raw, smoothed and normalized spectra in text files*
Experimental factors	*E. coli culture was treated by EOCs, chlorine, peroxide and tetracycline. Samples were harvested after 30, 60, 90 and 120 min.*
Experimental features	*E. coli culture at start of exponential phase was treated by EOCs in minimal inhibitory concentration. As reference conditions tetracycline 5 mg/L, NaClO 5 mg/L and hydrogen peroxide 3 g/L were used. The cells were harvested in 30, 60, 90 and 120 min after treatment. Cultures were resuspended in 1 mL of 70% ethanol in Eppendorf tube and then prepared to MALDI-TOF analysis according to Bruker standard procedure with formic acid extraction.*
Data source location	*Czech University of Life Sciences Prague*
Data accessibility	Data is with this article with additional data available at https://doi.org/10.17632/s294p9sf9r.1

**Value of the data**

• Low molecular weight proteins, which are not detectable by 2D GE, can be identified from MALDI-TOF spectra.
• Up- and down-regulated proteins in bacterial samples could be detected.
• The data can be used for comparison of low-molecular weight proteins of different bacteria to any change of environmental conditions.
• Protein expression profiles can be used to shed light on the mechanisms of inhibition of various agents and factors.

## 1 Data

**Fig. 1:**
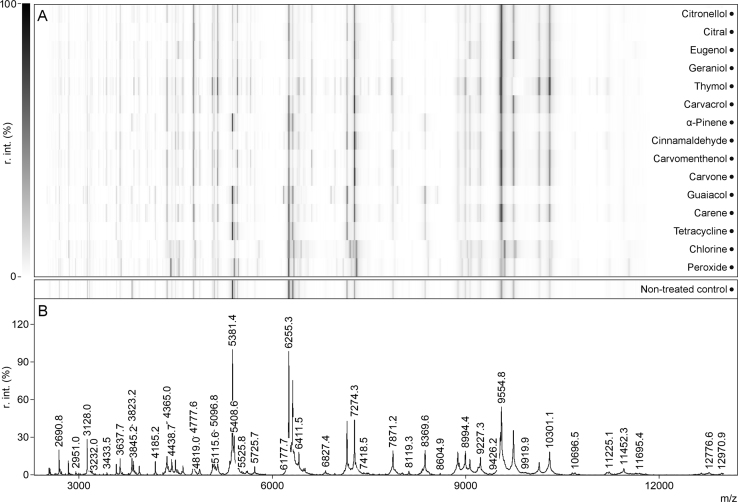
(A) Pseudo-gel views of mass spectra of treated samples used in this study collected 90 min after treatment. (B) Representative mass spectrum of *E. coli* (non-treated sample).

This data article contains the protein spectra obtained by MALDI-TOF from samples of *Escherichia coli*. Spectra are measured in *m*/*z* range from 1000 to 15,500 by Autoflex Speed (Bruker Daltonics, Bremen, Germany), flexControl 3.4 (Build 135) and modified MALDI Biotyper method (MBT_FC.par) was used (available on-line: http://dx.doi.org/10.17632/s294p9sf9r.1). Cultures of *E. coli* were treated with fourteen different substances (12 EOCs, peroxide, chlorine and tetracycline) and samples were harvested 30, 60, 90 and 120 min after treatment (Fig. 1).

## 2 Experimental design, materials and methods

### 2.1 Sample preparation

Microtitre plate wells were filled by 100 µl of LB broth – Lennox and then inoculated by fresh inocula prepared from *E. coli* overnight culture. The plates were incubated at 37 °C for 5 h, which corresponds with the end of the lag phase. The stock solutions of the active substances (Table 1) were prepared at concentration double MIC in the same broth. One hundred microliters of this solution was added to the *E. coli* culture. As reference the tetracycline 5 mg/L [2], NaClO 5 mg/L and hydrogen peroxide 3 g/L [3] were used. The cells were harvested in 30, 60, 90 and 120 min after treatment. Cultures were transferred from wells to Eppendorf tubes and mixed with 1 mL PBS (phosphate buffered saline). The suspension was centrifuged at 20 000×*g* for 2 min in Rotanta 450R (Hettich, DE) and supernatant was discarded. Then pellets were resuspended in 1 mL of 70% ethanol in Eppendorf tube. The cells were prepared to MALDI-TOF analysis according to Bruker standard procedure. Samples were centrifuged at 20 000×*g* for 2 min and supernatant was decanted. Two more minutes in centrifuge helped to remove residual ethanol from pellets by a pipette. Samples were left to dry for 30 min at room temperature to increase the extraction efficiency. Then, five microliters of 70% formic acid (Sigma-Aldrich, DE) was added to the pellet and mixed thoroughly by vortex. The same volume of ultra-pure acetonitrile (Fluka, DE) was added to the tube and mixed again. Finally, the samples were centrifuged at 20 000×*g* for 2 min and 1 μL supernatant was transferred on to a MTP 384 target plate ground steel BC (Bruker, DE). Each sample was placed on one spots around spot with calibrant (BTS, Bruker, DE). As soon as the sample spots have dried, the samples were overlaid with 1 μL HCCA (α-Cyano-4-hydroxycinnamic acid, Bruker, DE) matrix solution (acetonitrile 50%, water 47.5% and trifluoracetic acid 2.5%; concentration 10 mg of HCCA per millilitre). The sample spots were left to dry before analysis. MALDI-TOF analysis we carried out using an Autoflex Speed (Bruker Daltonics, Bremen, Germany), flexControl 3.4 (Build 135) and modified MALDI Biotyper method (MBT_FC.par) was used (available on-line: http://dx.doi.org/10.17632/s294p9sf9r.1). The ion source 1 was 19.38 kV, ion source 2 was 18.18 kV and detection was set from 1000 to 15,500 Da. All experiments were performed in independent biological triplicates for each agent and tested cultivation time. For each sample, three spectra were measured, and each spectrum was collected by 4000 shots in 200 steps.

**Table 1:** Concentrations of tested compounds against *E. coli* derived from minimal inhibitory concentrations.

Compound	CAS No.	Concentration	
		[µL L^−^^1^]	ID in dataset
Carvacrol	499-75-2	256	16
trans-Cinnamaldehyde	14371-10-9	256	20
Eugenol	97-53-0	512	8
(−)-Carvone	6485-40-1	1024	36
(+)-α-pinene	7785-70-8	1024	19
(±)-ß-Citronellol	106-22-9	1024	2
(1S)-(+)-3-Carene	498-15-7	1024	59
Thymol	89-83-8	1024	15
4-Carvomenthenol	562-74-4	2048	34
Citral	5392-40-5	2048	4
Geraniol	106-24-1	2048	9
Guaiacol	90-05-1	2048	55
Tetracycline	60-54-8	5 mg/L	ATB
Sodium hypochlorite	7681-52-9	5 mg/L	Cl
Hydrogen peroxide	7722-84-1	3 g/L	O
Control – non-treated culture			PC

### 2.2 Data processing

Public statistical R software, multiMS-toolbox and Mass Spectrum Analysis and Data Conversion Tool [4], [5] were used. Mass spectra of each sample treated by the tested agent from all three replications and measured dots were exported using FlexAnalysis 3.4 Compass 1.4 (Bruker Daltonics, Bremen, Germany). All the spectra were preprocessed using a self-tailored multiMS-toolbox 2.08 run in R software 3.4.2. All exported mass spectra were pre-processed by Savitzky-Golay smoothing filter and the best matched exponential baseline removed. The signal intensities were normalized to the same median of intensity ratios. For a protein identification and comprehensive discussion and of the full dataset, please see the research article that accompanies this data article (Božik et al., 2018) [1].
